# Contrasting Daytime Habitat Selection in Wild Red Deer Within and Outside Hunting Ban Areas Emphasises Importance of Small‐Scale Refuges From Humans

**DOI:** 10.1002/ece3.71407

**Published:** 2025-08-15

**Authors:** Thomas Rempfler, Wibke Peters, Claudio Signer, Flurin Filli, Hannes Jenny, Klaus Hackländer, Sven Buchmann, Pia Anderwald

**Affiliations:** ^1^ Department of Ecosystem Management, Climate and Biodiversity, Institute of Wildlife Biology and Game Management BOKU University Vienna Austria; ^2^ Swiss National Park Zernez Switzerland; ^3^ Swiss Federal Institute for Forest, Snow and Landscape Research Birmensdorf Switzerland; ^4^ Wildlife Biology and Management Research Unit Bavarian State Institute of Forestry Freising Germany; ^5^ Wildlife Management Unit Zurich University of Applied Sciences, Institute of Natural Resource Sciences Wädenswil Switzerland; ^6^ Hunting and Fisheries Department Canton of Grisons Chur Switzerland; ^7^ Deutsche Wildtier Stiftung Hamburg Germany

**Keywords:** behaviour, *Cervus elaphus*, human disturbance, integrated step selection functions, landscape of fear, protected area, wildlife management

## Abstract

1. Prey species such as red deer ( *Cervus elaphus* ) select their habitats according to their requirements for landscape features and adapt this selection to the presence of predators and humans. We tested how networks of different types of protected areas—the Swiss National Park (SNP) without hunting but with additional regulations for humans, and smaller‐scale hunting ban areas (all types together = HBAs)—influenced diurnal and nocturnal habitat selection in red deer compared with unprotected areas.
2. Using integrated step selection functions, we compared habitat selection of 243 GPS‐collared individuals from six study areas across the Central Alps during day and night, during the year and specifically during the short autumnal hunting season.
3. During the day, red deer avoided habitats where encounters with humans were likely, i.e., they selected for denser tree cover, greater distances to trails, steeper slopes, and for most of the year, for higher elevation. Importantly, in summer and autumn, they selected HBAs. At night, they showed the opposite selection. This daily pattern was absent in the study area centred on the SNP, where habitat selection was less specific overall. During the main hunting season, they selected HBAs over areas without protection during both day and night, and concurrently, habitat selection was less specific inside compared with outside HBAs.
4. HBAs allow red deer to select habitat largely independently of human impact. Accordingly, compensating habitat selection at night due to human disturbance during the daytime was observed in all study areas, except for the region centered on the SNP. Our results suggest that in human‐dominated landscapes, networks of small‐scale HBAs can support more natural habitat selection of the animals, especially when providing additional regulations to humans.

## Graphical abstract

The analyses of GPS locations have shown that in human‐dominated landscapes, networks of hunting ban areas allow red deer to select habitat largely independently of human impact.

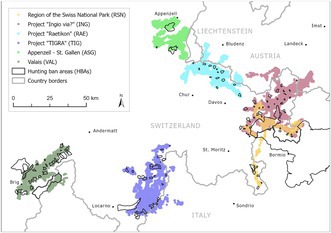

## 1 Introduction

Prey species are sensitive to predator presence and minimise risk exposure by modifying their behaviour (Brown et al. 1999). Besides innate behaviour (e.g., neonatal antipredator tactics; Atmeh et al. 2024), individuals assess risks by combining aspects of the predator (e.g., speed, size), their own physical condition (e.g., reproductive state, size) and environmental factors (e.g., time of day, amount of cover) based on experience and learning (Stankowich and Blumstein 2005).

The landscape of fear concept represents a heterogeneous distribution of relative levels of perceived predation risk and the associated level of fear a prey animal experiences in different parts of its home range (Laundré et al. 2010). Thus, this perception of risk is related to the physical landscape and predation risk, and results in accordingly adapted behaviour, for example, distribution patterns of prey and antipredator behaviour (Gaynor et al. 2019). Animal movement is the behavioural mechanism that links the multiscale process of habitat selection in response to biotic and abiotic factors (Johnson 1980). For example, elk ( *Cervus elaphus canadensis* ) shifted their habitat use and fed on lower quality forage in response to wolf reintroduction in Yellowstone National Park (Hernández and Laundré 2005). This concept is not only applied to natural predators but also in relation to humans, with hunting having direct and indirect effects on wildlife: in a landscape‐scale playback experiment, predators moved more cautiously when hearing human voices or became more elusive and reduced foraging activities (Suraci et al. 2019). Ungulates in turn modify their movements (Little et al. 2016) or feeding site selection to avoid hunters (Benhaiem et al. 2008). Female moose ( *Alces alces* ) that lost their young during the hunt stayed further away from settlements and at shorter distances from the forest the following year (Graf et al. 2024). Roe deer ( *Capreolus capreolus* ) modified their habitat use between day and night to avoid hunting (Bonnot et al. 2013). White‐tailed deer ( *Odocoileus virginianus* ) are also able to recognise local risks: on days following hunting from stands, the use of areas around the stands decreased during the day and increased at night (Sullivan et al. 2018).

Human activities can affect fitness (Shively et al. 2005) and even recreational activities can cause behavioural and physiological reactions in wildlife comparable to those in response to predation (Frid and Dill 2002; Stankowich 2008). These include increased vigilance (Beauchamp 2015), flight (Ydenberg and Dill 1986; Schnidrig‐Petrig and Ingold 2001), reduced activity levels (Graf et al. 2018) changes in habitat selection (Gander and Ingold 1997; Filla et al. 2017), reductions in parental investment (Gill et al. 2001) and effects on energy expenditure (Houston et al. 2012), resource acquisition, animal condition and finally reproductive success (Frid and Dill 2002). Over time and space, such individual effects can scale up to cumulative pressures at the population level (Sutherland 1996). Disturbance effects can be enhanced if humans are accompanied by dogs (Miller et al. 2001). On the other hand, during COVID‐19 lockdowns with reduced human mobility, spatial behaviour of wildlife changed, for example, to increased use of areas closer to roads and high human footprint, which indicates that animals reduced their avoidance of proximity to humans (Tucker et al. 2023).

Effects of human disturbance on the *Cervus* genus are relatively well studied (Mattioli et al. 2022). For example, Ciuti et al. (2012) suggested that effects on elk behaviour caused by human disturbance exceeded those of habitat and natural predators: human presence triggered increased vigilance and decreased foraging. Among food, topography and human activity, the latter has even been identified as the strongest driver of red deer movement (Mumme et al. 2023). Animals strongly respond to disturbance from human recreational activities by increasing their level of vigilance, but their response varies with the level of cover available, and they perceive hunting as a more acute threat than human recreation (Jayakody et al. 2008). Even red deer ( *Cervus elaphus* ) which appear to be habituated to regular disturbance within their home ranges, may alter their behaviour and avoid hiking trails (Sibbald et al. 2011; Westekemper et al. 2018). As human activity is mainly concentrated during the daytime, one avoidance strategy used by red deer—and other mammals (Gaynor et al. 2018)—consists in altering their diurnal behaviour, i.e., avoidance of humans during the day by using refuge areas and compensation by being more active at night (Godvik et al. 2009; Coppes et al. 2017).

Habitat selection of red deer during the green‐up season in a study in mountainous habitats depended, among other factors, on landscape characteristics and human presence: red deer commonly preferred shrub cover, flat terrain and lower to intermediate elevations, but avoided habitats with possible exposure to human activity, i.e., the vicinity of roads and trails, or areas far away from forest cover (Sigrist et al. 2022). The onset of the hunting season triggers fear reactions in red deer, i.e., increased flight distance, more time spent outside the core home range, and preference for dense vegetation, which may affect red deer distribution and harvesting efficiency (Meisingset et al. 2022). Reactions may differ somewhat between the sexes: for example, male red deer in Norway shifted their habitat preferences at the onset of the hunting season, while females did not, as they were already largely using cover when hunting started (Lone et al. 2015). In Canada, older female elk individually changed their behaviour as they aged and reduced movement rates to decrease the likelihood of encountering hunters (Thurfjell et al. 2017). In addition, they increased the use of secure areas (i.e., forest and steeper terrain) and adjusted their behaviour depending on the type of threat (bow and arrow vs. rifle hunters) (see also Proffitt et al. 2013). This fine‐tuning of elk behaviour to avoid hunters, as opposed to just becoming more cautious during the hunting season, highlights the behavioural plasticity of this species.

In the late 19th century, the first protected areas were created to conserve iconic landscapes and provide habitat for endangered wildlife (Watson et al. 2014). At the same time, when red deer in Switzerland were just returning after their extinction in the 18th century (Haller 2002), federal and cantonal wildlife reserves were originally designated in Switzerland with the intention to increase ungulate populations by banning hunting within these reserves. As the first large‐scale protected area in the Alps without any human use apart from restricted recreation, the Swiss National Park (SNP) was founded in 1914. The SNP provides the strongest degree of year‐round protection, and human disturbance is greatly reduced (category Ia protected area: Strict Nature Reserve). Inside all these types of hunting ban areas (HBAs), hunting is prohibited, while the SNP and federal wildlife reserves additionally protect wildlife from human disturbance, but at different levels. Authorities and managers in the Swiss Alps have supplemented this network with small‐scale HBAs, especially since the 1980s in order to manage the spatial distribution of red deer, as the species is known to use protected areas to avoid hunting activities (Haller 2002; Proffitt et al. 2010, 2013; Mikle et al. 2019). This approach is based on the expectation of the red deer's ability to reach these areas through seasonal migrations as summer habitats (Haller 2002), undertaken by parts of the populations (Fellmann 2022; Table S1).

While effects of larger protected areas on red deer are known, effects of networks of smaller‐scale HBAs have not been analysed yet. These networks offer an ideal experimental study design to compare behavioural adaptations inside versus outside protected areas. In this study, we tested (a) how red deer in six study areas in the Alps select habitats during day and night, as well as over the course of the year, and (b) how habitat selection specifically during the main hunting season differs inside and outside HBAs, at day and night, and between different study areas. We expected red deer to avoid humans during the day by selecting against habitat characteristics indicating human presence in all study areas. On the other hand, we expected no such avoidance at night, especially because there is no hunting at night. We further predicted (c) a selection for HBAs particularly due to hunting activity. These compensating patterns should be less pronounced in the study area which is centred on the SNP with its strict regulations for humans.

## 2 Materials and Methods

### 2.1 Study Areas

**FIGURE 1:**
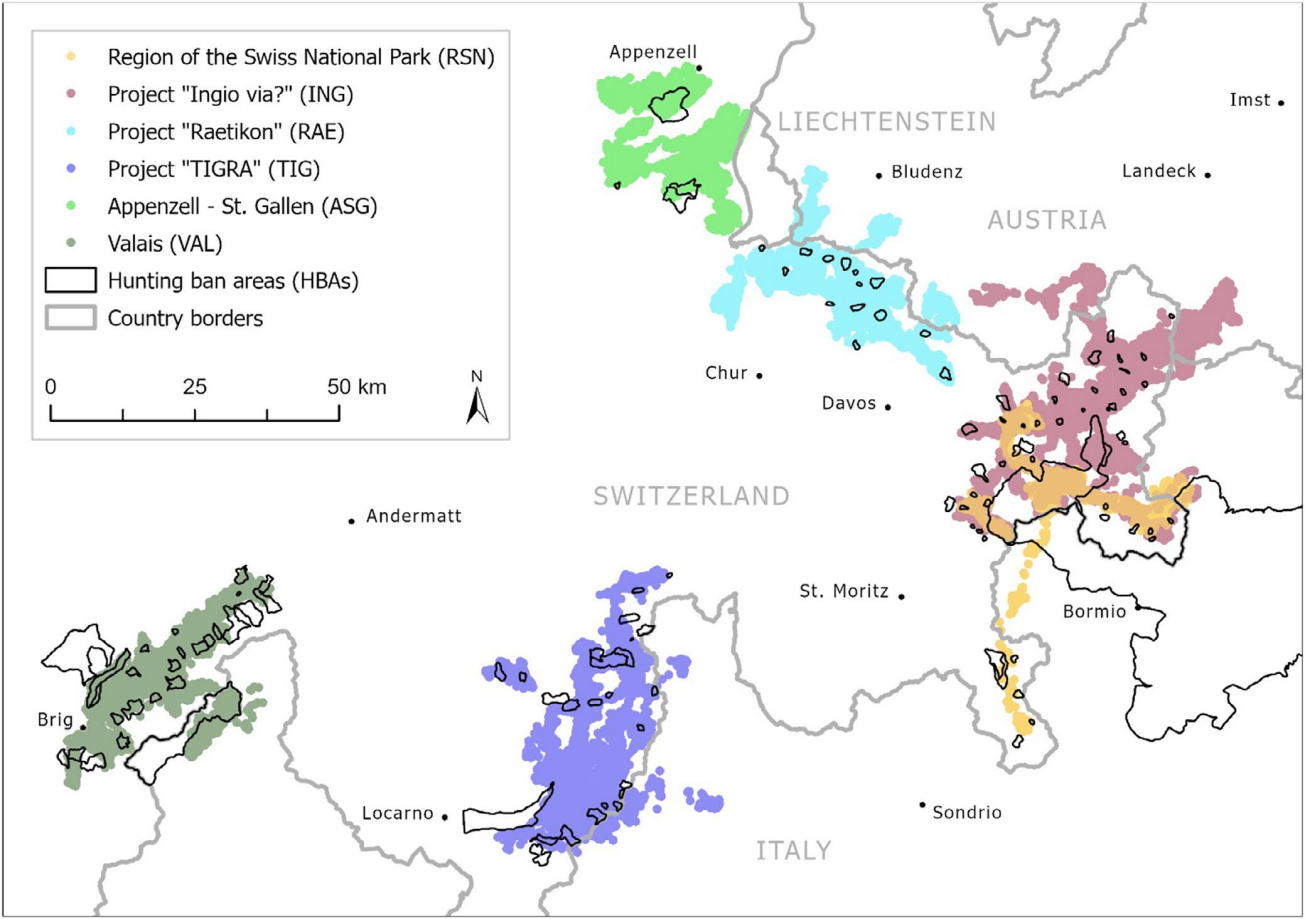
Study areas with red deer GPS locations (coloured locations) and HBAs (black polygons). Map and data: Hunting Departments of the cantons of Appenzell Inner‐Rhodes, Appenzell Outer‐Rhodes, Grisons, St. Gallen, Ticino and Valais, Principality of Liechtenstein, Swiss National Park, Vorarlberg Hunting Association, swisstopo. SNP 2024/09.

We used red deer GPS data from six study areas across the Alps: from western Austria, northern Italy, Liechtenstein, as well as eastern and southern Switzerland (Figure 1 and Table S1). Settlements are typically concentrated in the valley bottoms and recreation activity is generally high in all study areas. Major highways are mostly absent, except in the study area ‘TIGRA’ (TIG). The main agricultural land use are pastures with cattle and/or sheep. Hunting ungulates is generally permitted outside of HBAs according to regulations. In most of the Swiss study areas and the respective Italian parts, a licence‐based hunting system is practiced, where hunters can independently choose their place to hunt. In contrast, in the canton of St. Gallen, i.e., the main part of the study area ‘Appenzell – St. Gallen’ (ASG), as well as in Austria, hunting rights are linked to landownership, i.e., hunters are allowed to hunt only on specific hunting grounds (for details see Trouwborst and Hackländer 2018). Hunting in the study sites with licence hunting is mainly practiced for a period of 3 weeks in September, except for the study area ‘Valais’ (VAL; only 2 weeks in September, but can also include the first days of October), followed by additional hunting days in late autumn to fulfil hunting quotas. Hunting in the parts of the canton of St. Gallen in the study area ASG was practiced from mid August to mid December, in Austria and Italy from May to December. It is usually carried out from high or ground seats, without dogs, or stalking, occasionally also as drive hunts in small groups with few hunters.

In the SNP, all human activities are prohibited year‐round, except for hiking on designated trails during daylight hours between ca. June and November (depending on snow conditions). Further restrictions that serve to reduce disturbance to wildlife within the park include the exclusion of livestock, visitors not being allowed to bring dogs into the park (not even on a lead), and there is a ban on overflights including paragliding or flying drones. Any violations registered by National Park Rangers are heavily fined.

### 2.2 Red Deer Data

We analysed trajectories of 191 female and 118 male wild red deer that were captured and collared between 2010 and 2021. Except in Austria, there are no winter feeding stations. Individuals were immobilised with dart guns or captured in corral traps and anesthetised and equipped with GPS telemetry collars (VECTRONIC Aerospace GmbH). The collars recorded GPS locations for 1–3 years at a sampling rate of 1 or 3 h. Authorities and wildlife officials of the cantons executed the captures during winter (except for the study area of the ‘Region of the Swiss National Park’ (RSN), where animals were also captured in spring and early summer) in accordance with national animal welfare laws and under permits issued by the responsible bodies (GR1001411, RA 2009/2862‐6743_01, SG13‐12, GR2014‐07F, GR2015‐09, GR2017‐12F, GR2020‐08F, VS07‐17).

We subsampled GPS locations with the package ‘amt’ (Signer et al. 2019) to one fix every 3 h, which was the least common denominator over all studies, with a tolerance of 3 min. In addition, we removed the data for the first 3 days and the last day in order to exclude possible effects of the capture and the removal of the collar (Morellet et al. 2009; Jung et al. 2019). We then eliminated inaccurate locations using the screening method by Bjørneraas et al. (2010). To select individuals with access to HBAs, we estimated 99% Kernel density home ranges using the R package ‘adehabitatHR’ (Calenge 2022) based on all GPS locations of an individual, and overlapped them with the HBAs using the R package ‘sf’ (Pebesma et al. 2023). In case of an overlap, we included individuals with at least 80% fix rate success per month, resulting in a sample size of 243 individuals (Table S1).

### 2.3 Explanatory Variables

#### 2.3.1 Proxies of Human Presence

Within the study areas, there are different types of HBAs, which were available as geospatial vector data. Depending on their objectives, these designated areas restrict hunting as well as various other human activities, which may disturb wildlife or influence their behaviour (Grignolio et al. 2014). In the Swiss National Park (Strict Nature Reserve; 170 km^2^) all human use is prohibited except scientific study and hiking on trails. The Stelvio National Park (Protected Landscape; 1310 km^2^) is a category V protected area. Hunting is limited to a few areas, which are located outside of our analysis perimeter. The Swiss Federal Wildlife Reserves (14–26 km^2^) were originally intended to increase ungulate populations by spatial hunting bans. Nowadays, they primarily aim at protecting endangered species and habitats (§ 1 federal ordinance on Wildlife Reserves), while hunting is still banned. Furthermore, several Swiss cantons have implemented Cantonal Wildlife Reserves (0.15–12 km^2^) to spatially manage red deer distribution, among others the cantons of Grisons (§ 1 cantonal ordinance on Wildlife Reserves) and Valais (§ 35 cantonal hunting law). There are no HBAs within the study sites in Austria and Liechtenstein.

As the main parts of the study areas were situated in Switzerland, we used the road layer of the Swiss Topographic Landscape Model (TLM) as underlying data (Swisstopo 2015). Since we were interested in the nearest distances to trails, we only included trail categories of up to 2 m in width. For areas outside Switzerland, we used Protomaps (https://protomaps.com) to extract Open Street Map data for the project perimeter. We postedited lacking trails in ArcGIS Pro with Swisstopo's reference map 1:25′000 and combined it with the TLM. We calculated path distance from these trails, accounting for topography based on a digital elevation model with a grid size of 30 m (DEM; NASA 2020) in ArcGIS Pro (version 3.0.3, ESRI) to extract the distance values for all red deer locations in mountainous terrain, instead of planar distances.

#### 2.3.2 Landscape Variables

We used a DEM (NASA 2020) to derive elevation and slope (in degrees; R package ‘raster’ (van Etten et al. 2023)). We derived forest cover ranging from 0%–100% from the Copernicus Tree Cover Density product (Herrmann et al. 2017) by matching GPS data to the respective year of the tree cover density layer. We resampled all tree cover layers to a uniform resolution of 20 m because the respective products from 2012 and 2015 were only available at this resolution, while the product from 2018 was at a resolution of 10 m. All covariates and their expected relationships with red deer antipredator behaviour towards humans are summarised in Table 1.

**TABLE 1:** Covariates and their expected links to red deer antipredator behaviour towards humans.

Covariate	Type	Expected impact	Question	References
Tree cover density	Continuous	Selection for denser tree cover reduces the risk of being detected by humans (or hunters)	(a), (b), (c)	Lone et al. 2015; Meisingset et al. 2022; Sigrist et al. 2022
Distance to trails	Continuous	Selection for larger distances to trails reduces the risk to encounter humans	(a), (b), (c)	Sibbald et al. 2011; Westekemper et al. 2018
Slope	Continuous	Selection for steeper slopes reduces the risk to encounter humans	(a), (b), (c)	Thurfjell et al. 2017
Elevation	Continuous	Selection for higher elevation reduces the risk to encounter humans	(a), (b), (c)	
Hunting ban area	Factor (inside, outside)	Selection for HBAs reduces the risk of being hunted	(a), (b) as interaction with habitat covariates, (c) as interaction with hunting activity	Coppes et al. 2017; Mikle et al. 2019
Hunting activity	Factor (yes, no)	Increased use of HBAs due to hunting activities reduces the risk of being hunted	(c) as interaction with HBAs	Proffitt et al. 2010; Mikle et al. 2019
Step length	Continuous	(Not interpreted)	(a), (b), (c)	
Turning angle	Continuous	(Not interpreted)	(a), (b), (c)	

#### 2.3.3 Temporal Variables

Daytime was defined as the time between sunrise and sunset, nighttime including twilight as the opposite, with the R package ‘suncalc’ (Thieurmel and Elmarhraoui 2022).

The hunting season differed between study areas. While it lasts until December in Italy and Austria, it is limited to a few weeks in autumn in Switzerland. For analyses on the effects of hunting activity, we limited the spatial extent to Switzerland and to the period between August 15th and October 31st which spans over the period from before hunting started until after it ended.

### 2.4 Modelling Habitat Selection

In order to analyse habitat selection, we fitted integrated step selection functions (iSSFs; Avgar et al. 2016). All analyses were conducted with R version 4.2.0 (R Core Team 2022). Using the package ‘amt’ (Signer et al. 2019), we first calculated individual trajectories and then generated 10 random locations per observed location, based on movement‐related statistics (i.e., with a gamma distribution for step lengths and a Von Mises distribution for turning angles). We then extracted explanatory variables for end locations of each step and scaled all continuous variables. To account for individual‐specific variation in habitat selection, we fitted Poisson generalised linear mixed models (glmmTMB; Brooks et al. 2024; Muff et al. 2020) with random slopes per individual and year for each environmental variable, except turning angle (Webber et al. 2024). The intercept was estimated per stratum. To reduce potential bias caused by differences in movement patterns between individuals, we included the distance between two consecutive GPS locations (step length) and the cosine of the angular deviations (turning angle) in the models (Avgar et al. 2016; S2).

#### 2.4.1 Diurnal and Monthly Effects of Environmental Variables

For analyses of diurnal patterns, we fitted monthly step selection functions per study area separately for day and night to obtain an overview of monthly habitat selection. We next ran the same models, but separated by sex, and subsequently compared only females because no data from males was available in the study area RSN. We then pooled all study areas (pooled study areas = PSA) except for RSN, as similar trends were detected across explanatory variables in the area‐specific models with the exception of RSN. In order to correct for multiple testing (*n* = 264 for all study areas separately, and *n* = 48 for PSA vs. RSN) we applied a Holm–Bonferroni correction to *p* values in the model outputs (Holm 1979).

#### 2.4.2 Effects of Hunting Ban Areas During the Main Hunting Season

To test for the effects of HBAs on red deer habitat selection specifically during the hunting season, we selected only September data, i.e., the main hunting season in all study areas. Exploratory analysis revealed similar habitat availability inside and outside HBAs. We included interaction terms between HBAs and all environmental variables in the model except for turning angles. Again, we first analysed each study area separately for day and night, and then pooled all study areas (PSA), except for RSN.

#### 2.4.3 Effects of Hunting Ban Areas due to Hunting Activity

Finally, we tested whether red deer selected HBAs specifically due to hunting activity or whether their selection was simply seasonal. Thus, we included an interaction term between HBA and hunting activity in the model.

## 3 Results

### 3.1 Diurnal and Monthly Effects of Environmental Variables

**FIGURE 2:**
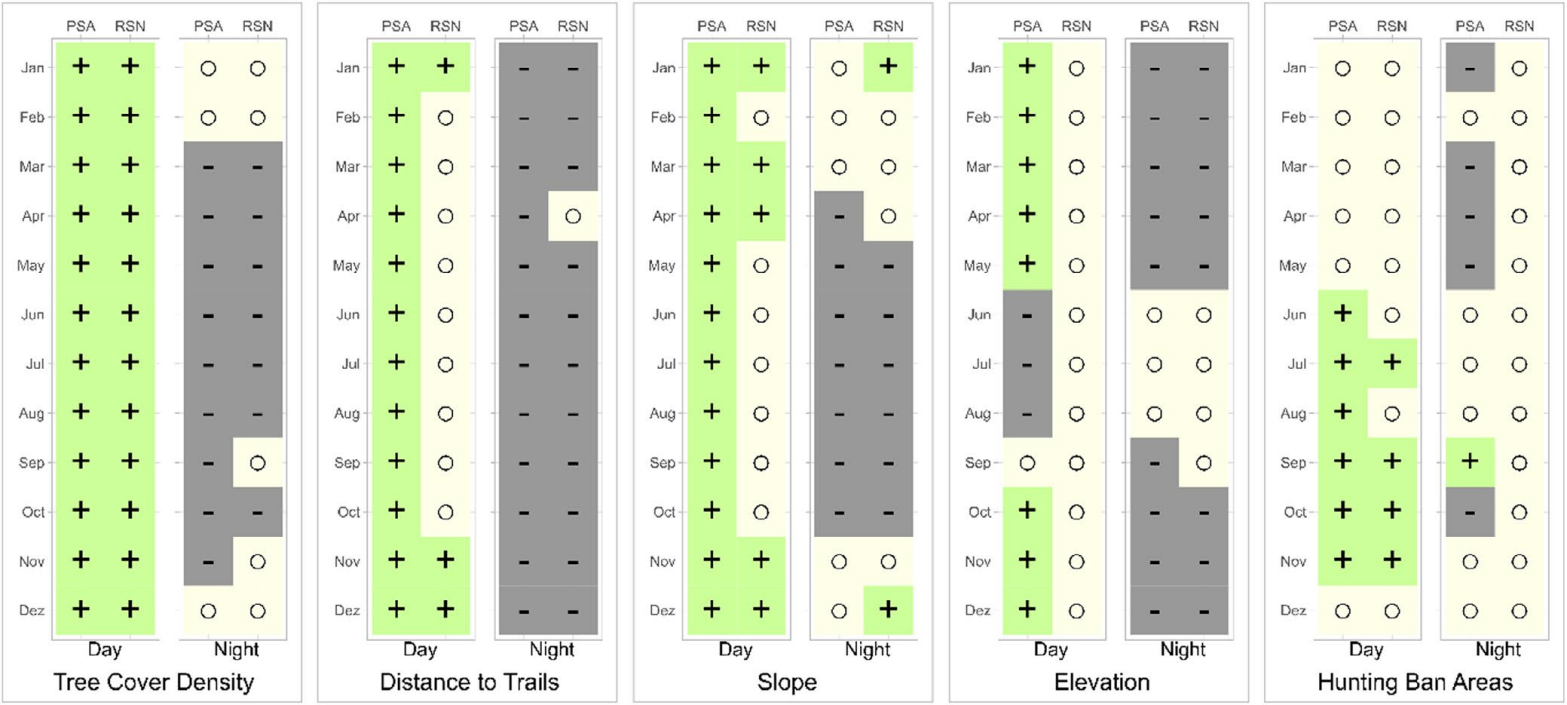
Monthly effects of each environmental variable (tree cover density, distance to trails, slope, elevation, hunting ban areas), included in the habitat models for female red deer. Models were run separately for day and night, for the pooled study areas PSA (ING, RAE, TIG, ASG and VAL), and RSN with individual‐years as random effects (green plus = significant positive, grey minus = significant negative effect, yellow circle = nonsignificant effect after Holm–Bonferroni correction).

Red deer selected contrasting habitats during the day than at night, but less consistently so in RSN than in PSA (Figure 2 and Table S3). During the day, red deer in PSA selected for higher tree cover density, longer distances to trails, and for steeper slopes all year round. Except for summer, they selected for higher elevation. In summer and autumn, they selected for HBAs. At night, they showed the opposite pattern, i.e., they selected lower tree cover density, shorter distances to trails, flatter slopes and lower elevation. They only selected for HBAs in September nights. Effects were absent for tree cover density and slope during winter nights and for elevation during summer nights. During summer and autumn nights, they selected for flatter slopes.

Unlike in PSA, red deer in RSN generally showed less contrasting habitat selection between day and night. During the day, they selected longer distances to trails and for steeper slopes only in winter, and not at all for elevation (Figure 2 and Table S4). The selection at night was similar to PSA. Effects showed little difference between the sexes (Figure S5).

### 3.2 Effects of Hunting Ban Areas During the Main Hunting Season

**FIGURE 3:**
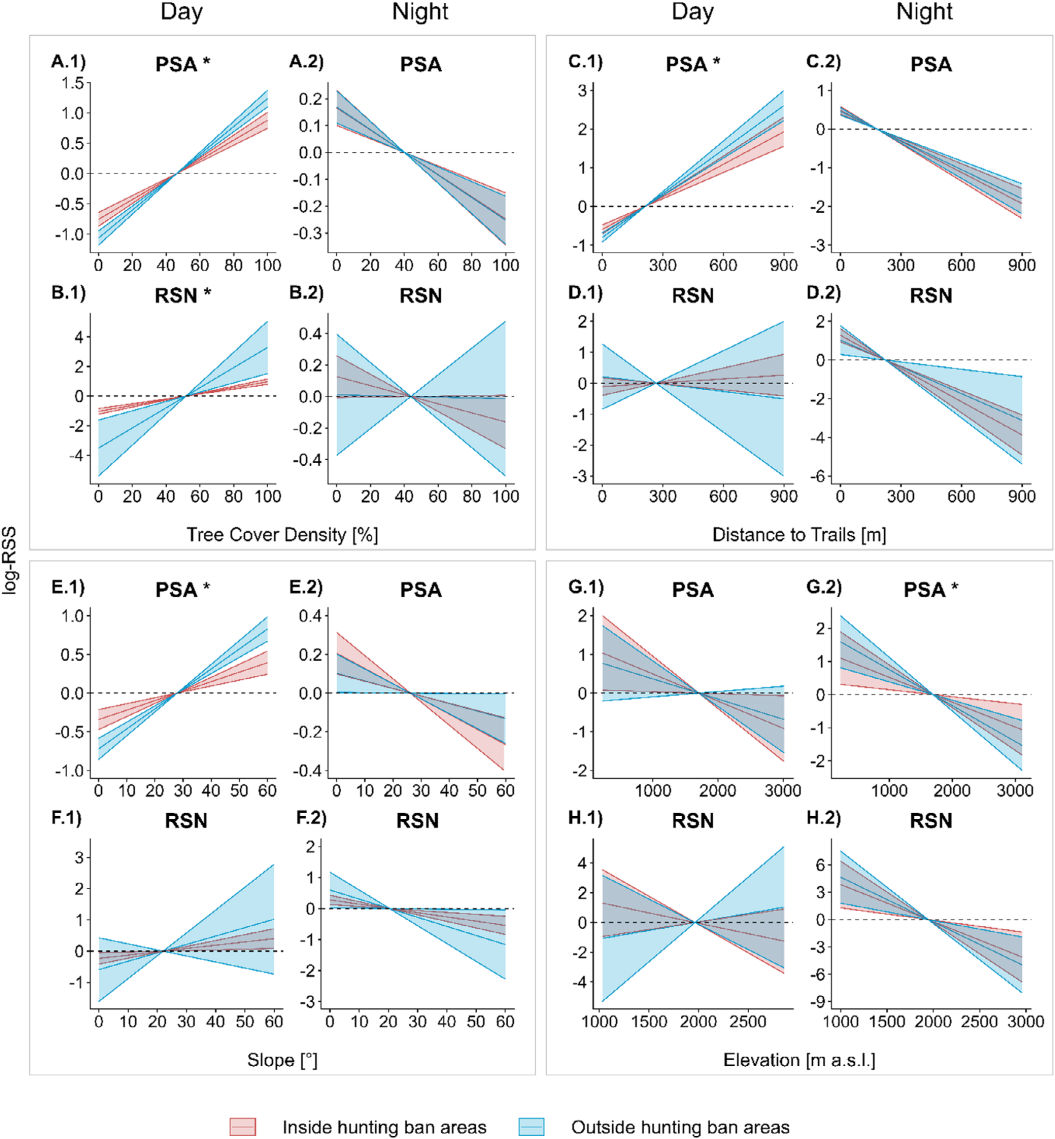
Effects of HBAs on habitat selection concerning tree cover density (A, B), distance to trails (C, D), slope (E, F) and elevation (G, H), per study area during day and at night in September (red = inside HBA, blue = outside HBA). Log‐RSS values were calculated relative to the average habitat in the study area based on a step selection analysis. * = significant difference in effect direction between inside and outside HBAs. Shaded areas encompass all pointwise 95% confidence intervals.

The comparison of habitat selection inside and outside HBAs is most meaningful during the main hunting season, which in all study areas is in September. With the protection from hunting, habitat selection by red deer differed inside and outside HBAs and between day and night (Figure 3 and Table S6; Figure S7). In all study areas, red deer selected HBAs to hunted areas during the day and at night. In PSA, coefficients had the same directions inside and outside HBAs. During the day, red deer selected for denser tree cover (Figure 3A.1), greater distances to trails (Figure 3C.1), and for steeper slopes (Figure 3E.1). This selection during the day was significantly weaker inside than outside HBAs. At night, red deer showed the opposite pattern of habitat selection, i.e., they selected for lower tree cover density (Figure 3A.2), shorter distances to trails (Figure 3C.2), and for flatter slopes (Figure 3E.2). This selection at night did not differ between inside and outside HBAs.

Red deer in RSN showed less specific habitat selection than in PSA. Inside HBAs, they did not select for any covariates during the day, except for denser tree cover. This selection was stronger outside HBAs (Figure 3B.1). At night, red deer selected for shorter distances to trails inside and outside HBAs (Figure 3D.2).

### 3.3 Effects of Hunting Ban Areas due to Hunting Activity

Interactions between HBAs and hunting activity in autumn for PSA showed that female red deer did indeed increase their use of HBAs due to hunting activity during the day and at night (after Holm–Bonferroni correction; Table S8). In contrast, in RSN there was a significant effect at night whereas no significant interaction was detectable during the day (Table S8) when the animals showed a selection for HBAs already (see Figure 2).

## 4 Discussion

### 4.1 Diurnal and Monthly Effects of Environmental Variables

Integrated step selection functions for all study areas except RSN consistently yielded monthly red deer habitat selection patterns that differed between day and night. During the day, their selection for dense forest cover and steep terrain, areas inside HBAs and further from trails indicated avoidance of areas closer to humans (Figure 2 and Figure S3–S5). These results are consistent with findings by Godvik et al. (2009), Sibbald et al. (2011) and Sigrist et al. (2022). However, they were in stark contrast to red deer habitat use at night, when the animals selected for low tree cover density, which corresponds to very low values for open areas, flat terrain and short distances to trails, but hardly chose HBAs. These contrasting patterns of habitat selection compared with those observed during the day suggest compensation in the relative safety of darkness. In agreement with Godvik et al. (2009), Coppes et al. (2017) and Gaynor et al. (2018), red deer in PSA thus avoided habitats where encounters with humans were likely during daytime but used them at night. This pattern could be encouraged by the ban on night hunting in Switzerland.

Red deer in the study area RSN, which was centered on the SNP, behaved differently from all other study areas (Figure 2 and Figure S5). Namely, they showed no significant year‐round avoidance of habitat characteristics that could be attributed to human disturbance during the day, except for tree cover density. The strict protection measures from human disturbance in the SNP, and particularly the predictability of human presence on trails (i.e., guaranteed absence elsewhere), are the most likely explanation why red deer in RSN during summer and autumn neither kept large distances from hiking trails nor retreated to steep terrain and high elevations during daytime, as they did in PSA. Habituation of red deer to predictable movement of humans along designated trails versus a sensitivity to off‐trail hiking has been shown in an experimental setting by Westekemper et al. (2018). Contributing factors to habituation of elk in North America have been consistent and predictable human behaviour, but also high densities, prohibited hunting, and habitats that provide winter range (Thompson and Henderson 1998). In the absence of mortality risk from hunting by humans, as in our HBAs, animals can also learn to exploit human‐disturbed areas by desensitising and eventually habituating to human stimuli (Bejder et al. 2009). To some prey species, areas frequented by humans serve as refuges from predators that are less inclined to habituate to human presence (Shannon et al. 2014). This human shield effect was observed, for example, in the Yellowstone Ecosystem, where moose birth sites were located close to paved roads, which brown bears avoided (Berger 2007). On the other hand, human‐habituated individuals could become bolder and thus more vulnerable to predation (Geffroy et al. 2015). In the absence of natural predators, the hunting ban in combination with the restriction of visitors to hiking trails in the SNP likely amplified the differences in habitat use compared with the other study areas. Interestingly, these differences lasted for much of the year (Figure 2), suggesting that red deer in RSN avoided humans less in spring when large parts of the population—due to seasonal migration—were in their winter habitats outside the SNP (Haller 2002). Presumably, these red deer have learned that humans do not pose a risk outside the hunting season.

Seasonal differences in habitat selection of red deer both in PSA and RSN indicated that in winter, animals saved energy during the day and did not compensate at night (see also Arnold et al. 2004; Pépin et al. 2009). They reduced forage intake in winter and thus avoided expending energy on the unproductive search for more scarcely available food (Arnold et al. 2015). During winter nights, they did not select low tree cover density (i.e., open areas in the extreme) or flat slopes. The selection for HBAs was restricted to summer and autumn, likely to generally avoid human disturbances. This is in line with a study in Germany where red deer used refuge and core zones more frequently than border zones during summer (Coppes et al. 2017). In addition, they benefited from undisturbed rutting activities in autumn (Frid and Dill 2002), and avoided hunting (Mikle et al. 2019).

### 4.2 Effects of Hunting Ban Areas During the Main Hunting Season

During the main hunting season in September, red deer showed a clear selection for HBAs (Table S6). Effects of habitat selection patterns for both inside and outside HBAs had the same direction, but with contrasting directions between day‐ and nighttime (Figure 3). Besides the general avoidance of humans during the day, red deer avoided humans more strongly outside HBAs, as hypothesised.

The pattern of a less clear habitat selection in RSN remained after the subdivision into inside and outside HBAs (Figure 3). This can be explained by the lack of a need to select for habitat parameters associated with human avoidance within the SNP. As our results show that compensation at night is not necessary in this study area, we conclude that the SNP as the center of our study area RSN best fulfills its purpose in terms of reducing effects of human disturbance.

### 4.3 Effects of Hunting Ban Areas due to Hunting Activity

Previous findings state that red deer in autumn migrate due to the onset of hunting (Rivrud et al. 2016) and seek protected areas particularly at this time of year (Mikle et al. 2019). The significant positive interaction between hunting activity and the use of HBAs for PSA during day and night, and for RSN at night (Table S8; for this analysis only inside Switzerland) indicates that more cautious habitat selection of red deer due to hunting activity extends even into the hours of darkness. We interpret this result as a direct response to hunting activities, especially because nighttime was defined as the time from dusk until dawn in our study. During the day, however, hunting activity had no additional effect on the use of HBAs in RSN, likely because the animals already showed a significant selection for HBAs during summer and autumn anyway. Another explanation for the selection of HBAs in autumn even at night may also be a selection for undisturbed rutting sites (Frid and Dill 2002). As the main hunting season in Switzerland coincides with the red deer rut, the two effects are difficult to disentangle.

In order to further refine analyses of compensating behaviour, a next step would be to compare movement behaviour, behavioural states and activity patterns during day and night, inside and outside HBAs.

## 5 Management Implications

The differences in habitat selection during the day and at night, as well as inside and outside HBAs, corroborate previous findings that red deer in a human‐dominated landscape are able to adapt their spatiotemporal behaviour to human activity (Ciuti et al. 2012; Mumme et al. 2023). We have shown that their selection against habitat characteristics indicating human presence depends on the time of day. We have further shown that HBAs—even at small scales—are a promising tool in red deer management. By offering spatiotemporal refuge habitats, managers in Switzerland take advantage of the capacity of red deer to recognise a landscape of fear. Increased use of lower tree cover density during the day increases red deer visibility. In a hunting for fear approach, hunting induces sufficiently strong risk effects to induce behavioural adaptations (Cromsigt et al. 2013). Consequently, we reason that especially the combination of short intense hunting periods with HBAs may lead to predictable red deer behaviour and can facilitate regulation. Thus, in human‐dominated landscapes, we suggest that networks of small‐scale HBAs that connect red deer habitats may aid in decreasing hunting pressure and maximising harvest efficiency (see also Griesberger et al. (2022)), especially when providing additional regulations to other forms of human use, such as restricting tourism.

## Author Contributions

**Thomas Rempfler:** conceptualization (lead), data curation (lead), formal analysis (lead), investigation (equal), methodology (equal), resources (equal), validation (equal), visualization (lead), writing – original draft (lead), writing – review and editing (equal). **Wibke Peters:** conceptualization (supporting), formal analysis (supporting), investigation (equal), methodology (equal), resources (equal), validation (equal), writing – review and editing (equal). **Claudio Signer:** conceptualization (supporting), data curation (equal), investigation (equal), resources (equal), validation (equal), writing – review and editing (equal). **Flurin Filli:** conceptualization (lead), data curation (equal), funding acquisition (lead), investigation (equal), project administration (equal), resources (equal), supervision (supporting), validation (equal), writing – review and editing (equal). **Hannes Jenny:** conceptualization (lead), data curation (equal), funding acquisition (lead), investigation (equal), project administration (equal), resources (equal), validation (equal), writing – review and editing (equal). **Klaus Hackländer:** conceptualization (supporting), investigation (equal), resources (equal), supervision (supporting), validation (equal), writing – review and editing (equal). **Sven Buchmann:** conceptualization (supporting), investigation (equal), resources (equal), validation (equal), visualization (lead), writing – review and editing (equal). **Pia Anderwald:** conceptualization (supporting), formal analysis (lead), investigation (equal), methodology (equal), resources (equal), supervision (lead), validation (equal), writing – original draft (equal), writing – review and editing (equal).

## Conflicts of Interest

The authors declare no conflicts of interest.

## Supporting information


Data S1.

## Data Availability

All data used in this manuscript are available via https://www.parcs.ch/snp/mmd_fullentry.php?docu_id=53933.
