# Correction to ‘Contrasting Daytime Habitat Selection in Wild Red Deer Within and Outside Hunting Ban Areas Emphasises Importance of Small‐Scale Refuges From Humans’

**DOI:** 10.1002/ece3.72104

**Published:** 2025-09-07

Rempfler, T., W. Peters, C. Signer, et al. 2025. “Contrasting Daytime Habitat Selection in Wild Red Deer Within and Outside Hunting Ban Areas Emphasises Importance of Small‐Scale Refuges From Humans.” *Ecology and Evolution* 15, no. 6: e71407. https://doi.org/10.1002/ece3.71407.

Due to a production error, the following funding information was omitted from the Acknowledgements section: Open access funding provided by Universität fur Bodenkultur Wien/KEMÖ.

The online version of this article has been corrected accordingly.

We apologise for this error.

