# Supplementary material for: Contrasting Daytime Habitat Selection in Wild Red Deer Within and Outside Hunting Ban Areas Emphasises Importance of Small‐Scale Refuges From Humans
Source: Ecol Evol. 2025 Aug 15;15(8):e71407. doi: 10.1002/ece3.71407 (PMC12355003; doi:10.1002/ece3.71407)
Supplement: Supplementary file 1 — Data S1. [file ECE3-15-e71407-s001.docx]

## Appendix

#### Table S1: Study areas

Table S1. Study areas with start and end year of the studies, the number of red deer individuals after filtering (ID), the number of hunting ban areas per study area (HBA), mean temperatures in January and July, respectively, mean precipitation, and the mean number of days per year with snow cover. Means are taken over the study years (MeteoSwiss, 2022).

The study areas cover montane to alpine regions in a) inner alpine valleys (studies “Region of the Swiss National Park” (RSN) and “Ingio via” (ING), the Swiss National Park with its surrounding areas in the canton of Grisons, Tyrol, the Autonomous province of Bolzano – South Tyrol and the province of Sondrio, and study “Valais” (VAL) in the canton of Valais and the province Verbano – Cusio – Ossola), in b) the northeastern Swiss Pre-Alps and Alps (study “Appenzell – St. Gallen” (ASG) in the cantons of Appenzell Inner-Rhodes, Appenzell Outer-Rhodes and St. Gallen and study “Raetikon” (RAE) with Liechtenstein, Vorarlberg and the canton of Grisons), and in c) the alpine south (study “TIGRA” (TIG) in the cantons of Grisons and Ticino and provinces of Como, Lecco and Sondrio). Long-term weather station data indicate differences in climate between the study areas (MeteoSwiss, 2022). Cold, long winters and little precipitation are characteristic for the inner alpine study areas ING, RSN and VAL. In contrast, precipitation is abundant and winters are shorter in the study areas ASG and RAE on the north side of the Alps, and in TIG on the south side.

#### S2: Model structures for hypotheses a), b) and c)

a) Diurnal and monthly effects of environmental variables

model_a <- glmmTMB(case_ ~ -1 +

*# environment*

elevation + slope+

*# human*

hunting_ban_area + tree_cover_density + distance_trail +

*# movement*

step_length + cos_turning_angle +

*# stratum*

(1 | step_id) +

*# random slopes “id_year”*

(0 + elevation | id_year) + (0 + slope | id_year) + (0+hunting_ban_area | id_year) +

(0 + tree_cover_density | id_year) + (0 + distance_trail | id_year) +

(0 + step_length | id_year),

family = poisson, data = data_scaled, doFit = TRUE,

start = list(theta=c(log(1e3), rep(0,6))),

map = list(theta=factor(c(NA, 1:6))),

control = glmmTMBControl(parallel = 35))

b) Effects of hunting ban areas during the main hunting season

model_b <- glmmTMB(case_ ~ -1 +

hunting_ban_area*(elevation + slope+ tree_cover_density + distance_trail +

step_length) + cos_turning_angle +

*# stratum*

(1 | step_id) +

*# random slopes “id_year”*

(0 + elevation | id_year) + (0 + slope | id_year) + (0 + tree_cover_density | id_year) +

(0 + distance_trail | id_year) + (0 + step_length | id_year),

family = poisson, data = data_scaled, doFit = TRUE,

start = list(theta=c(log(1e3), rep(0,5))),

map = list(theta=factor(c(NA, 1:5))),

control = glmmTMBControl(parallel = 35))

c) Effects of hunting ban areas due to hunting activity

model_c <- glmmTMB(case_ ~ -1 +

hunting_ban_area + hunting_ban_area:hunting_activity + elevation + slope +

tree_cover_density +distance_trail + step_length + cos_turning_angle +

*# stratum*

(1 | step_id) +

*# random slopes “id_year”*

(0 + elevation | id_year) + (0 + slope | id_year) + (0 + tree_cover_density | id_year) +

(0 + distance_trail | id_year) + (0 + step_length | id_year),

family = poisson, data = data_scaled, doFit = TRUE,

start = list(theta=c(log(1e3), rep(0,5))),

map = list(theta=factor(c(NA, 1:5))),

control = glmmTMBControl(parallel = 35))

#### Table S3

Table S3. Monthly model outputs of glmmTMB of the pooled study areas PSA (* = significant effect after Holm-Bonferroni correction).

#### Table S4

Table S4. Monthly model outputs of glmmTMB of the study area RSN (* = significant effect after Holm-Bonferroni correction).

#### Figure S5: Monthly effects of habitat variables per study area.

| 1. **Females** | 1. **Males** |
| --- | --- |


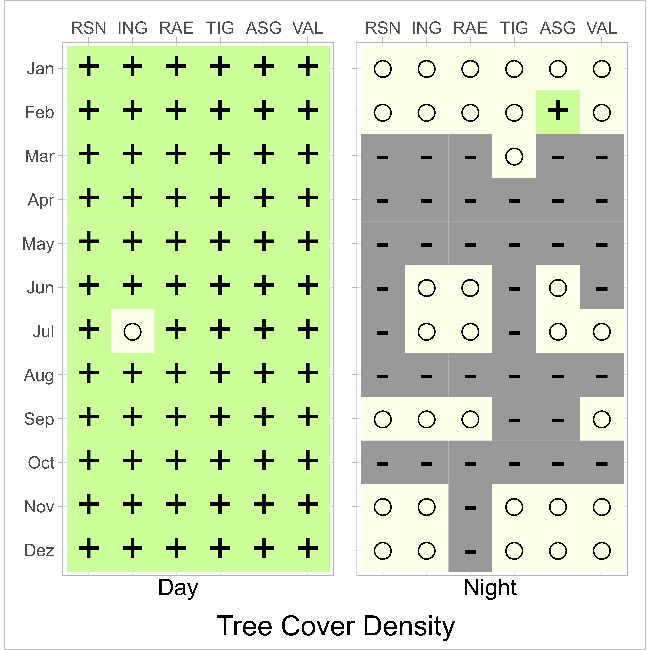

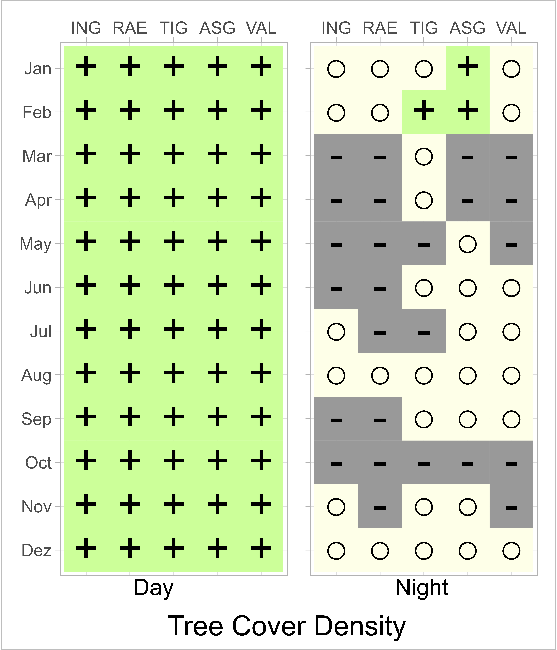

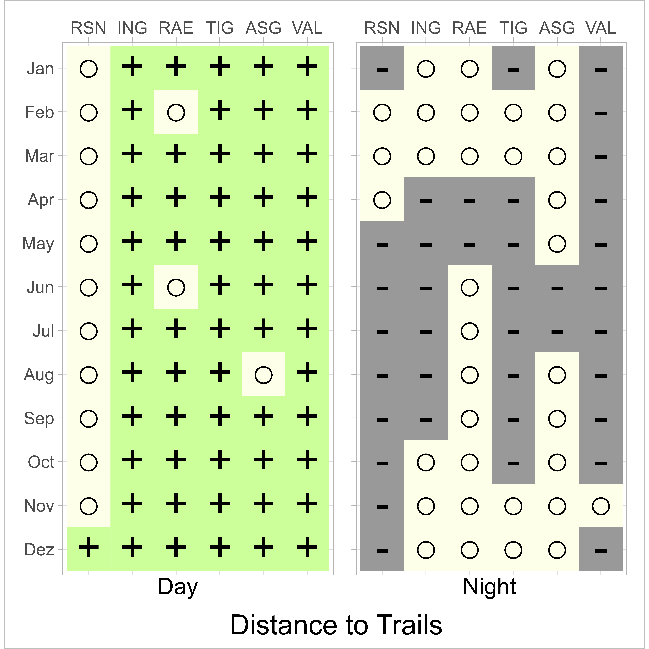

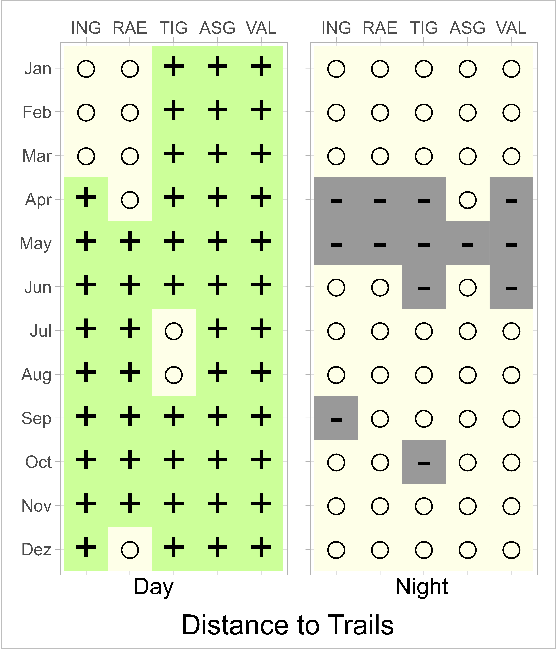

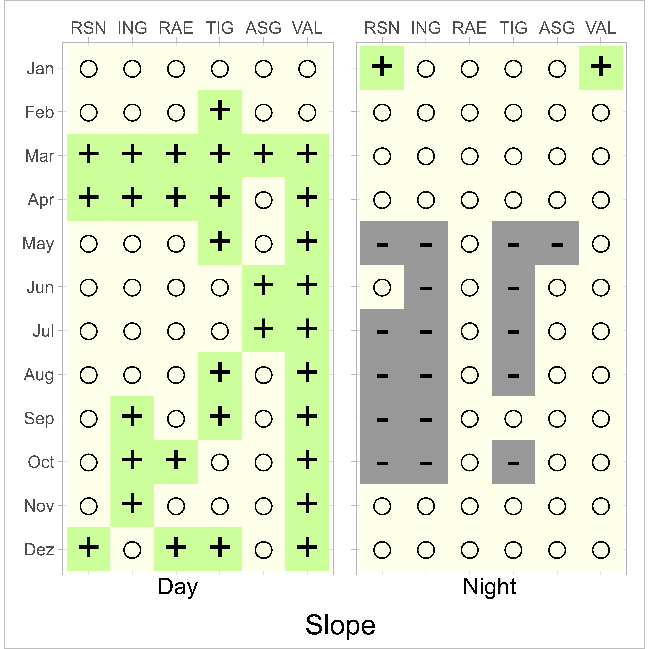

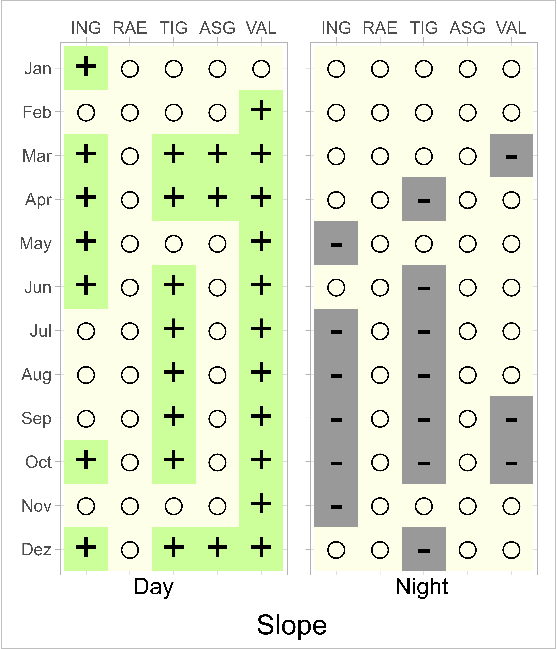

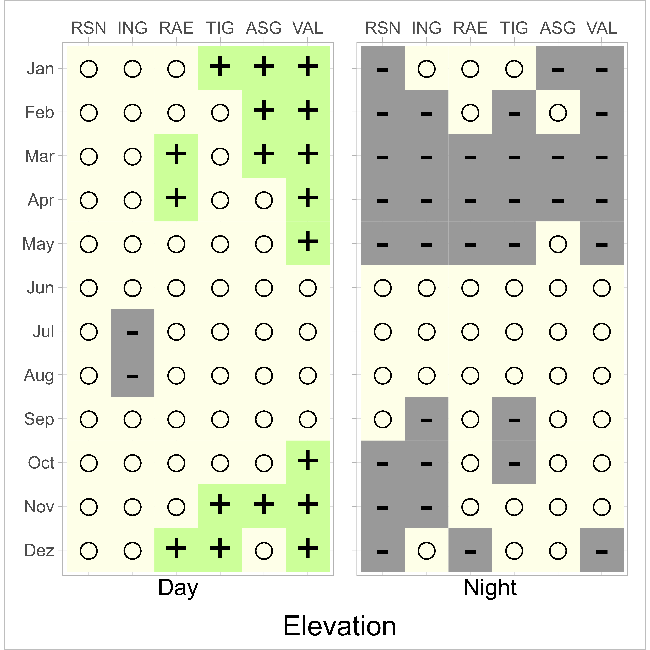

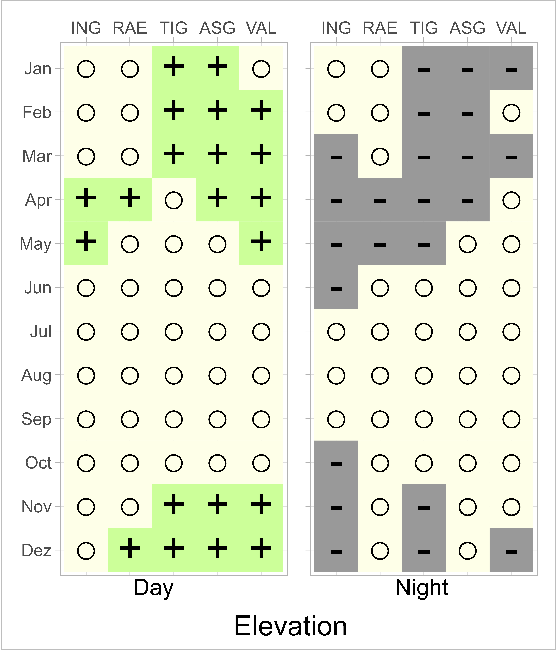

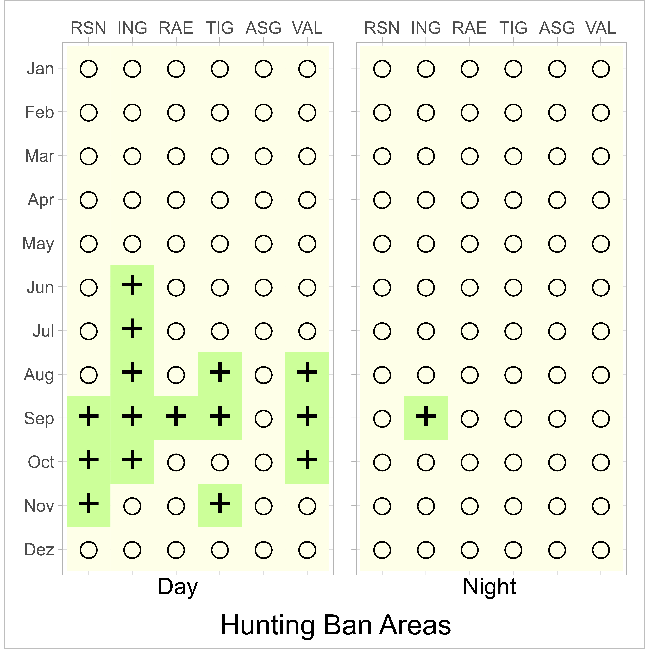

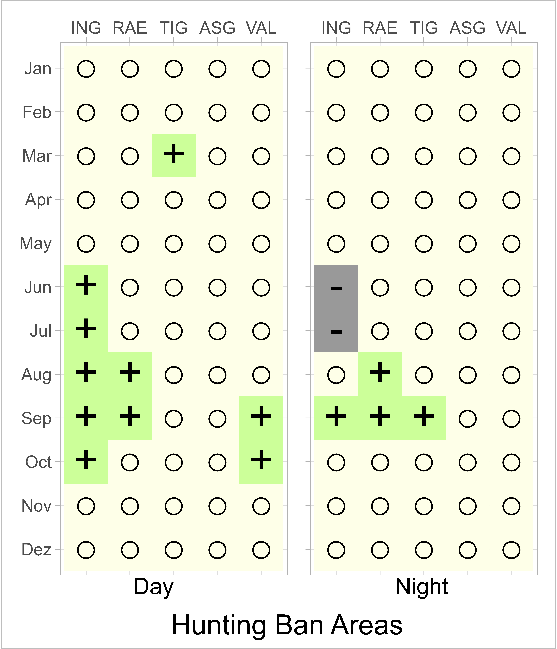


| 1. **Females** | 1. **Males** |
| --- | --- |

Figure S5. Monthly effects of each habitat variable (tree cover density, distance to trails, slope, elevation, and hunting ban areas), included in the habitat models for red deer. Models were run separately for females (A) and males (B), for day and night, and for each study area with individual-years as random effects (green plus = significant positive, grey minus = significant negative effect, yellow circle = non-significant effect after Holm-Bonferroni correction). Please note that there was no GPS data on males from the study area RSN (B).

#### Table S6

Table S6. Model outputs of glmmTMB with the interaction of HBAs and habitat parameters during day and at night for PSA and RSN (* = significant effect after Holm-Bonferroni correction).

#### Figure S7: Interactions of the habitat variables in September, separately for each study area.


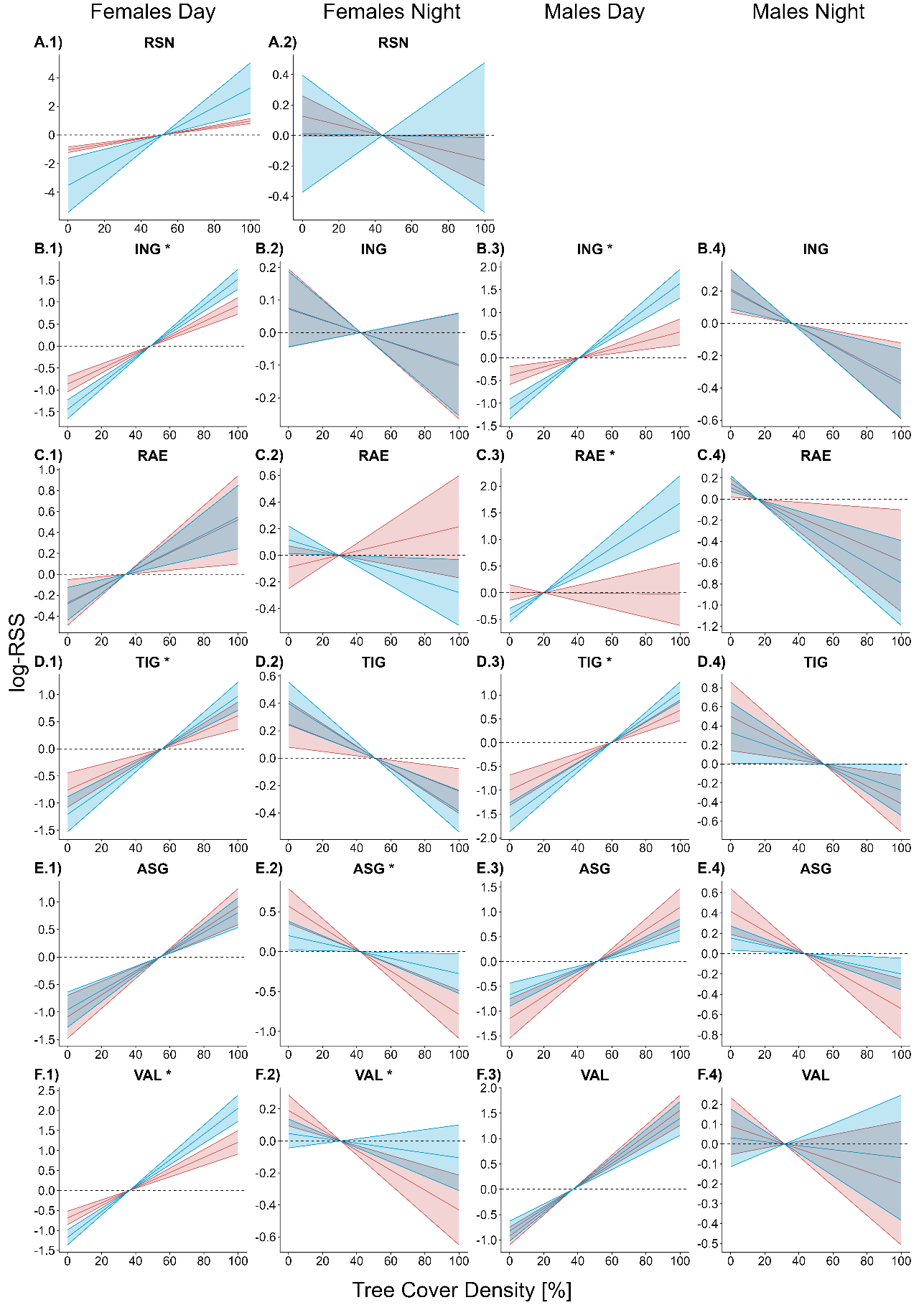


Figure S7a. Effects of HBAs on habitat selection concerning tree cover density, per study area during day and at night in September (red = inside HBA, blue = outside HBA). Log-RSS values were calculated relative to the average habitat in the study area based on a step selection analysis. * = significant difference in the effect direction between inside and outside HBAs. Shaded areas encompass all pointwise 95% confidence intervals.


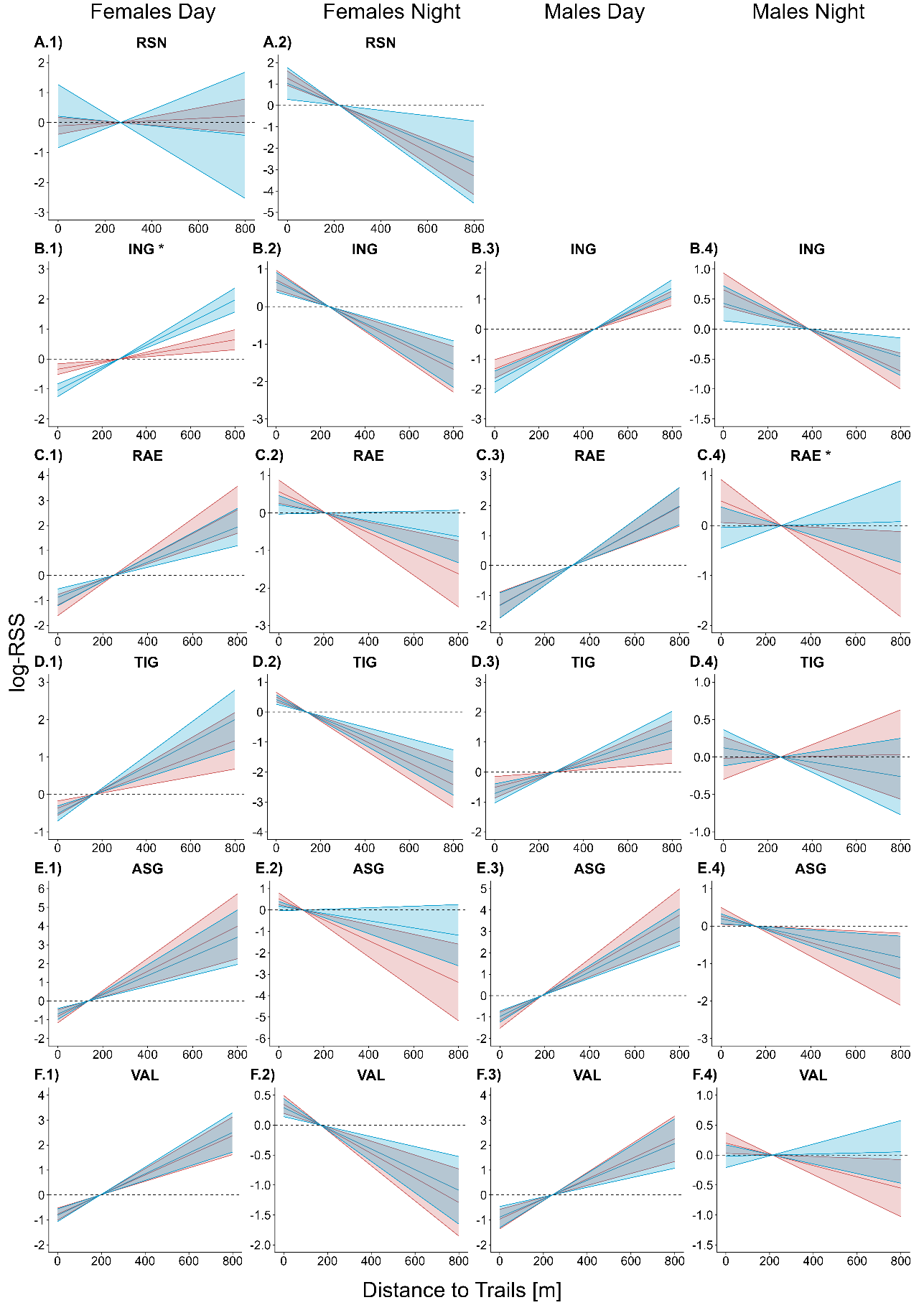


Figure S7b. Effects of HBAs on habitat selection concerning distance to trails, per study area during day and at night in September (red = inside HBA, blue = outside HBA). Log-RSS values were calculated relative to the average habitat in the study area based on a step selection analysis. * = significant difference in the effect direction between inside and outside HBAs. Shaded areas encompass all pointwise 95% confidence intervals.


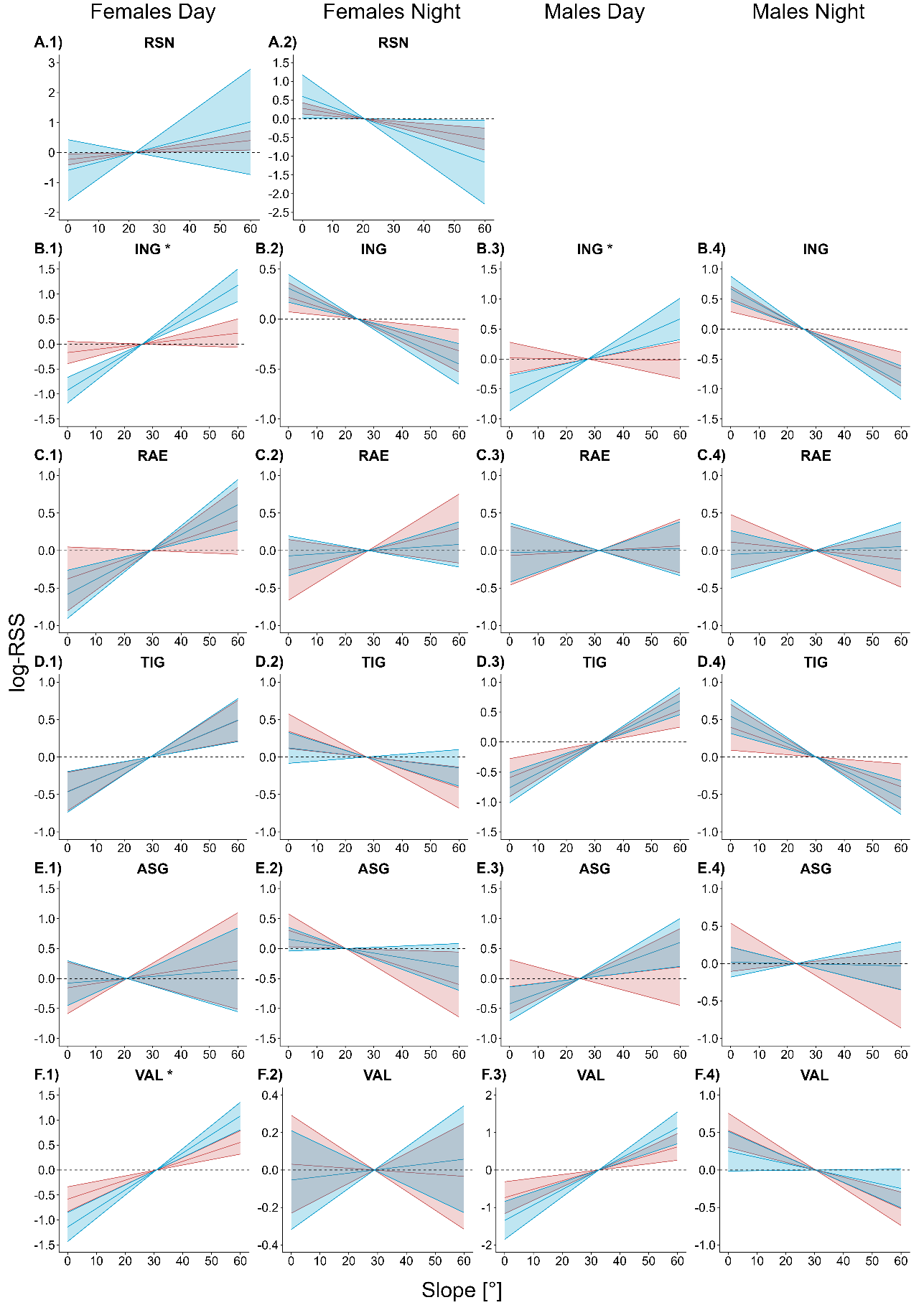


Figure S7c Effects of HBAs on habitat selection concerning slope, per study area during day and at night in September (red = inside HBA, blue = outside HBA). Log-RSS values were calculated relative to the average habitat in the study area based on a step selection analysis. * = significant difference in the effect direction between inside and outside HBAs. Shaded areas encompass all pointwise 95% confidence intervals.


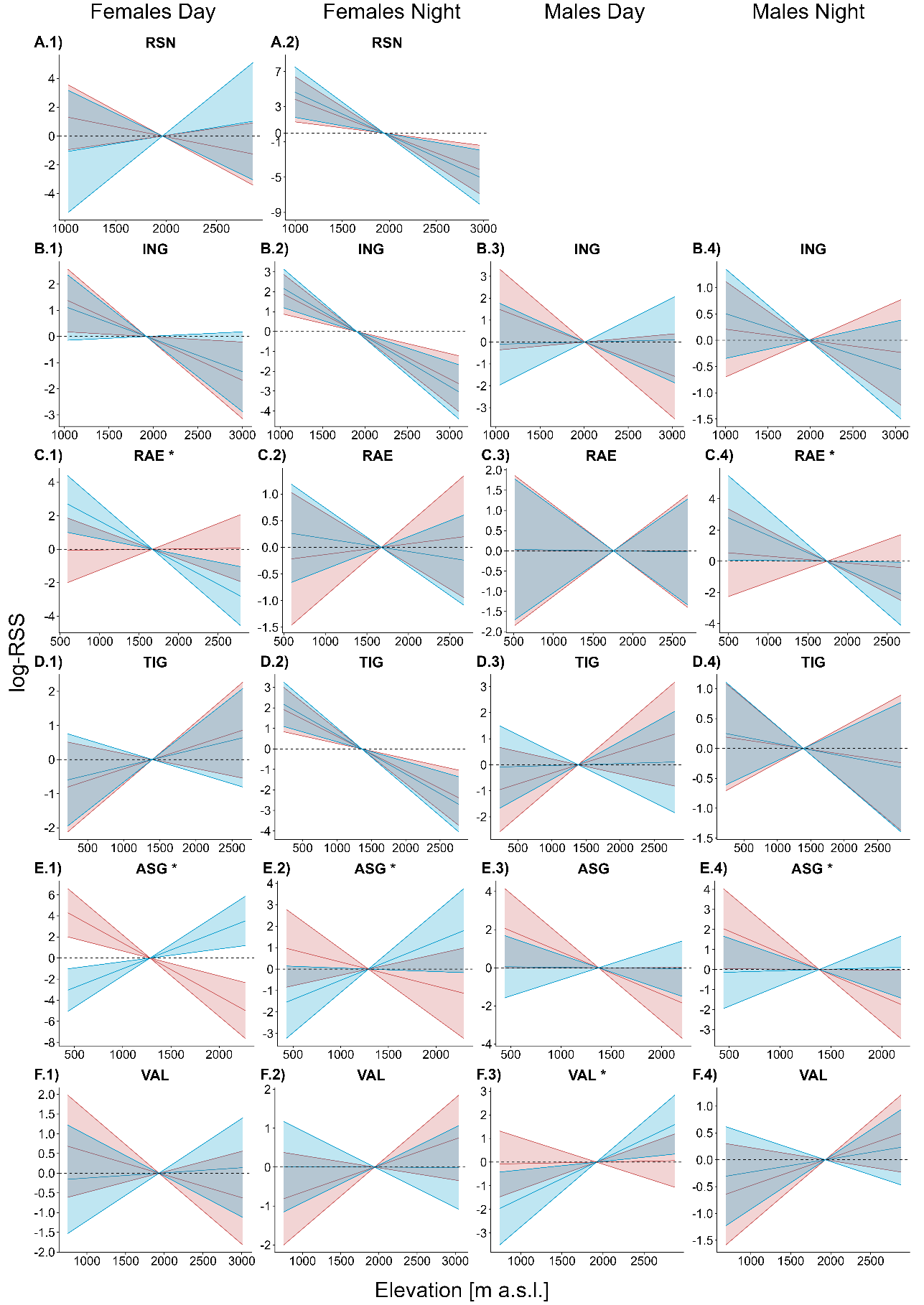


Figure S7d. Effects of HBAs on habitat selection concerning elevation, per study area during day and at night in September (red = inside HBA, blue = outside HBA). Log-RSS values were calculated relative to the average habitat in the study area based on a step selection analysis. * = significant difference in the effect direction between inside and outside HBAs. Shaded areas encompass all pointwise 95% confidence intervals.

#### Table S8

Table S8. Model outputs of glmmTMB with the interaction of HBAs and hunting activity in Switzerland during day and at night for PSA and RSN (* = significant effect after Holm-Bonferroni correction).
